# A Case of Double Consciousness, with Remarks by Professor Caldwell

**Published:** 1846-09

**Authors:** B. F. Berkley

**Affiliations:** Moorfield, Ohio


ART. II.—*A Case of Double Consciousness, with Remarks by Professor Caldwell.* By B. F. BERKLEY, M.D., of Moorfield, Ohio, in a letter to Professor C.

JUNE 18TH, 1846.

*Dear Sir:—*

Knowing that you have paid much attention to the science of mental philosophy, I have taken the liberty to trouble you with an imperfect report of the most extraordinary case of mental disease, perhaps, ever recorded; at least so far as I am informed in the history of our profession.

Mrs. N. B., a citizen of this county, living about sixteen miles from Cadiz, the county seat, a married lady, aged 39 years, has been the subject of neuralgia of the face for about 17 years, otherwise a healthy woman. About five or six years ago she grew worse, (previously having attracted no particular attention) the disease assuming a strictly periodical type, returning every two weeks—at which times she suffered the most excruciating agony in the course of the fifth pair of nerves of the right side of the face.. After suffering two or three hours in this way, she not unfrequently became sick at the stomach, and would vomit and purge. All these symptoms after a while subsiding, she would become entirely insensible to all external impressions. In this situation she would commence preaching in a loud and clear voice, and continue from two to three hours. She would then sink down as if she had fainted, and in fifteen or twenty minutes awake without the least knowledge of what had transpired.

She has had these periodical spells of preaching for five or six years every two weeks regularly, never having missed but two or three times. The novelty of the case has of course attracted a great many strangers from all parts of the country, and every body had something to say in regard to the matter—some alleging that it was all deception—some that the woman was divinely inspired—others, that she possessed as many devils as Mary Magdalene.

Hearing so many, and such contradictory statements, I determined to visit her myself, not believing much of what I had heard. Accordingly on last Sunday, the 15th inst., her regular day, I visited her. I determined to be there early in the morning (her preaching hour being at 11 o'clock, A.M.) to see her both before and after performance. I arrived at the house at 9 o'clock, and to my gratification found I was the first that had arrived; no person being present but myself and the family, I was introduced to Mrs. B—— by her husband in a private room. She was sitting in an arm chair, suffering all the agony of a severe attack of facial neuralgia of the right side, though somewhat different from most cases of that disease. There was no twitching of the muscles, great turgescence of the vessels of the face and neck; muscles of the neck very rigid; eyes very red; excessive intolerance of light, so much so, that she could scarcely bear to elevate the eyelids.

She says she feels an almost insupportable weight, like an incubus upon her head; there is an abundant secretion of saliva, which is altogether from the right side of the mouth. I talked with her for about an hour, or as long as she was capable of talking. I found her a very intelligent woman; she wished to know if there was nothing that would relieve her. I asked her if she had undergone any medical treatment. She said she had; that several eminent physicians had given her medicine. She had been cupped, her head shaved and blistered, ointment of veratria applied to the course of the nerve, and all the most noted antiperiodics, such as arsenic, the preparations of iron, &c., given in succession without the least benfit. She thought that under the tonic treatment she had got worse.

She continued to get worse and worse from the time I went into the room until about 11 o'clock, when her eyes closed and she became perfectly insensible to external impressions. In this situation she commenced talking.

She was placed in the sitting posture in a large room where a great number of strangers had collected. When she first commenced talking she appeared to be choked with a frothy saliva—but she soon cleared her throat, and preached for two hours and ten minutes in a clear and distinct voice—sufficiently loud to be heard a hundred yards. She commences in the form of a prayer, but soon changes to preaching, quoting scripture very fluently and giving explanations. Sometimes she appears to wander in her mind, and not place her words properly, though this is seldom the case. Sometimes her appeals would be the most pathetic and eloquent, I ever heard. The first warning you have that she is about to conclude is the free spitting up of this frothy saliva. As soon as that appears she falters and falls over. She continues insensible for fifteen or twenty minutes, all the time spitting up this saliva, when she awakes by yawning like a person who had been asleep, and looks about with a vacant stare. She soon however regains her senses, and looks like another person, and knows nothing of what has transpired.

The most remarkable circumstance connected with this case is, that she can neither see, hear, nor feel, during all the time she is preaching. She is not disturbed by any noise that may be made, and if pricked with any sharp instrument, does not flinch, and her eyes are closed during the whole time.

It is the opinion of some of the physicians who have visited her that a limb might be amputated without her knowledge. After she recovers from this singular situation all the redness of the face and eyes disappears; she resumes her natural appearance and seems cheerful, and says her pain has vanished. I would remark that it is several hours before she can walk, as she seems to have no use of her limbs—but the next day she resumes her ordinary domestic duties with as much vigor as any of her neighbors, until the next paroxysm.

This may all be called deception or a religious fanatacism; but I think the history of the woman, and every circumstance connected with the case, seem to preclude such an idea. In the first place, it has been a well-marked case of neuralgia for many years; she is not very loquacious, not so much so as most of her sex, never was an enthusiast on any subject; belongs to the Presbyterian church, and never was known to have a desire to preach, or talk in public on any subject. Taken all together it is one of the most extraordinary cases of disease of the nervous system that has ever occurred, and wholly inexplicable by me, and every one else that has seen her.

Her family is among the most respectable in the county; and I have conversed with men who have known the woman from a child, and none except a few uncharitable and ignorant persons, from whom she differs in doctrine, have ever accused her of trying to deceive the community.

*Remarks.*—The foregoing is one of those morbid affections to which the term MYSTERIOUS is almost *uniformly* and with much affected *significancy* and *wisdom* attached, not only by the great *mystifying body* of laymen, clergymen, and pseudo-philosophers, but even by the medical profession itself. Yet, if by the term we mean to designate something "*above human comprehension*," (and such is its most correct interpretation) there is no more *mystery* connected with it, than there is with a paroxysm of fever and ague, an attack of peripneumonia, or even with a sound and comfortable sleep. We can, I mean, just as easily comprehend, and as intelligibly explain the *double consciousness* and *somniloquence* of Mrs. B. as we can either of the other three conditions of the human system to which I have referred. The reason is plain. As far as *true and immediate causation* is concerned, we are essentially ignorant of them all. And so are we indeed of every other vital phenomenon—more especially if it be seated in the nervous system. For, entirely ignorant as we are of the genuine action, both healthy and morbid, of the other tissues of the body, we are, if possible, seemingly still more so of the action of the nerves.

Until within the last fifty years, scarcely any one, except enthusiasts and imposters, pretended to any real knowledge of the nervous system, beyond that of its mere existence and appearance—more particularly of its *central organ, the brain.* Dr. Gall, by his discovery of PHRENOLOGY, toward the close of the last century, first brought that organ (the *chef d'œuvre* of the Deity on earth) from the gloom of midnight to the glimmer of morning. And scarcely has our light in relation to it advanced as yet beyond that glimmering. As respects *practical purposes* this will not be denied, whatever may be said or thought in relation to theory.

The affection of Mrs. B., on which I am offering these remarks, belongs to the class of diseases, our knowledge of which is believed to have been most improved by the discovery of Dr. Gall. The principal maladies of that class are, insanity in its different forms, epilepsy, somnambulism, somniloqence, (of which Mrs. B.'s complaint is but a modification) St. Vitus's dance, hysteria, tetanus, hydrophobia, and a few other like affections—especially those denominated *mesmeric.*

I am aware that, in classing mesmeric somniloquence with that which is called *natural*, the complaint of Mrs. B., I come into collision with the sentiments of a distinguished writer, expressed in an article, sometime since delivered, as an address, and subsequently published by him, on that subject. But, when supported by facts, such collision cannot move me. And, were the present a suitable occasion for discussing the question, many indubitable facts might be adduced to show, that *natural* and *mesmeric*, somniloquence not only belong to the *same* CLASS of affections, but that they are veritably *modifications* of the *same* affection. Nor do I consider it at all certain that I shall receive from physicians a *general* or even an *extensive* concurrence in the opinion, that the complaint I am considering is very closely allied to insanity. Yet, to my mind, no position can be more clear—nor any opinion better founded and more satisfactory. But I must no longer dwell on this view of the subject, but proceed to one more practically useful.

The veiy respectable author of the foregoing article has solicited my opinion in relation to the most effectual mode of treating Mrs. B.'s complaint.

Though this is a question both delicate and entangled—especially as the patient has been attended by several able physicians, and acted on by powerful remedies for periodical complaints—and, *more* especially inasmuch as the lady has never been visited by me. Still, however, as nothing administered has proved beneficial to her, a prescription shall be hazarded. And that prescription is, that, provided she be sufficiently susceptible of the influence, (as I think it highly probable she is) let Mrs. B. be thrown into a profound mesmeric sleep, about two hours before the time of the paroxysm's commencement, and allowed to remain in it at least *four or five hours.* By the "commencement of the paroxysm" I mean the onset of *the neuralgia*, which appears to be the *prelude* to the *attack of somniloquism.* If *the former* therefore be prevented, so will the *latter*—precisely as the prevention of the *chill* of an intermittent prevents the subsequent elements of the fit.

It *may* and probably *will* be necessary for Mrs. B. to remain in the mesmeric sleep more than five hours. She ought to be thrown into it at least two hours before the first symptoms of neuralgia appear, and continue in it until two hours after the time at which it turns to somniloquism. The mesmeriser moreover ought to be expert and powerful, and the sleep as deep and perfect as it can be made. This remedy may not *effect a cure* on the first, nor even perhaps on the second and third trials. If the sleep, however, can be produced, it will at first give relief, and by subsequent and careful repetitions ultimately *cure.* But, if negligently and imperfectly employed, it will, like other remedies, under like circumstances, be useless and perhaps hurtful.

Am I asked how mesmeric sleep is to prevent neuralgia and its sequelæ, in the case of Mrs. B.? I answer, by taking such thorough and stable possession of the whole nervous system, as to render it entirely insensible, and proof against every other sort of impression. And this I know it does. Hence, during its continuance, teeth have been extracted, luxated and fractured bones reduced and set, tumors excised, and limbs amputated, without exciting in the patients a shade of sensation. Some of these operations, where there was no excitation of feeling I have myself witnessed. I was also a spectator of the following experiment performed on a lady in this city (*Louisville.*)

The poles of a very powerful electro-magnetic apparatus being placed in her hands, and the machine put in action, she could not tolerate the impression of the aura, for a single moment, but dropped the poles with a start and a slight scream. Being an uncommonly susceptible mesmerizee, she was then deeply mesmerised, and the poles again placed in her hands, and the machine much more highly excited than before. And she was as insensible to the aura as a marble statue. Nor could any person present, in his natural condition, bear the thrilling of it for an instant. I myself grasped the poles, and was compelled to drop them again, as suddenly as the lady had previously done.

But, for my belief in the efficacy of mesmerism as a remedy for periodical neuralgia I have yet other reasons, which most persons will deem still sounder and stronger than those I have rendered. In several slight cases and in one very severe one, I have witnessed its complete success. Nor, where the sleep can be rendered sufficiently deep, am I prepared to believe that it will ever fail.

